# Screen time and its association with psychological wellbeing and sleep quality: overview of the Fujian Adolescent Mental Wellness Study (FAMWeS)

**DOI:** 10.3389/fpsyg.2026.1686399

**Published:** 2026-09-11

**Authors:** Yulan Lin, Jian Jiang, Zhijie Luo, Qingying Wang, Fengqin Zou, Haridah Alias, Li Ping Wong, Zhijian Hu

**Affiliations:** 1 Fujian Key Laboratory of Environmental Factors and Cancer, Department of Epidemiology and Health Statistics, School of Public Health, Fujian Medical University, Fuzhou, Fujian, China; 2 Department of Emergency, Fuzhou University Affiliated Provincial Hospital, Fuzhou, Fujian, China; 3 Centre for Population Health (CePH), Department of Social and Preventive Medicine, Faculty of Medicine, Universiti Malaya, Kuala Lumpur, Malaysia; 4 Department of Medicine, College of Medicine, Korea University, Seoul, Republic of Korea

**Keywords:** anxiety, depression, screen time, sleep quality, stress

## Abstract

**Background:**

The objective of this cross-sectional observational study was to examine the structural associations between demographic, family-related, psychological, and sleep-related factors and screen time among adolescents in the Fujian Adolescent Mental Wellness Study (FAMWeS).

**Method:**

A cross-sectional survey was conducted among secondary and high school students in Fujian Province, China, from May to July 2023. Primary outcomes included overall screen media use, emotional state (assessed by the Depression, Anxiety, and Stress Scale for Youth, DASS-Y), and sleep quality (assessed by the Pittsburgh Sleep Quality Index, PSQI).

**Results:**

Analysis of 48,432 complete responses revealed that 36.5% of participants reported a daily screen time of 30 min to 1 h, while 11.3% reported more than 4 h per day. The partial least squares structural equation modeling (PLS-SEM) indicated that screen time was positively associated with PSQI global score (β = 0.116, *p* < 0.001), depression (β = 0.047, *p* < 0.001) and stress (*p* = 0.075, *p* < 0.001) scales. Study grade exhibited a significant inverse relationship with screen time (β = −0.092, *p* < 0.001). Higher screen time was significantly correlated with older age (β = 0.069, *p* < 0.001) and male gender (β = −0.072, *p* < 0.001). Furthermore, both paternal (β = −0.056, *p* < 0.001) and maternal (β = −0.059, *p* < 0.001) education levels were significantly inversely related to screen time.

**Conclusion:**

The study identified significant associations between higher screen time, poorer sleep quality, and elevated depression and stress levels, highlighting the importance of promoting healthy screen-use behaviors and supporting adolescents’ sleep quality and psychological well-being.

## Introduction

The rapid expansion of digital technology has led to a significant rise in screen time among adolescents. Adolescents are particularly susceptible to the impacts of extended screen exposure due to their developmental stage that involves complex changes that make them particularly vulnerable to adverse outcome across their life course (Povey et al., 2022). Research in Canada indicates that adolescents spend over 7.5 h per day on various screens, with 20% spending 5 h or more per day on social media alone (Boak et al., 2018; Katapally et al., 2018). The Canadian 24-H Movement Guidelines recommend that children and adolescents limit daily screen time to no more than 2 h (Tremblay et al., 2016; World Health Organization, 2020). An earlier study conducted in China from 2016 to 2017 found that the average daily screen time of Chinese primary and secondary school students was 1.65 h (Cheng et al., 2023). A more recent study from 2019 found that only about a quarter of children and adolescents adhered to the screen time guideline of no more than 2 h per day (Ke et al., 2023).

According to a systematic review and meta-analysis, in Chinese school-aged children and adolescents, screen time was most strongly associated with psycho-behavioral problems (81.8%), followed by myopia (59.2%), and adiposity (50.6%) (Zhang et al., 2022). Several recent studies have also reported that high screen time is associated with mental and behavioral problems among Chinese children and adolescents (Song et al., 2020; Wang X. et al., 2023). The Chinese government recognizes the adverse impact of technology overuse and has implemented measures to combat adolescent technology addiction (China Law Translate, 2021). China’s legal framework for protecting minors involves a combination of cybersecurity, data protection, and specific protections for minors. By enforcing these laws and regulations, the Chinese government seeks to minimize the risks of internet use among minors while enhancing its educational and developmental benefits. Psychological health issues associated with screen time are not exclusive to China but are a worldwide concern. A review of studies from various countries worldwide, including those conducted in China, reported that screen time has been associated with mental health problems in adolescents (Santos et al., 2023).

Screen time, sleep quality, and psychological well-being are closely interrelated, although the direction of these relationships remains complex and likely bidirectional. Although many previous studies have focused on the potential adverse effects of excessive screen time on sleep quality and psychological well-being, the relationships are likely to be bidirectional. Emerging evidence suggests that adolescents experiencing psychological distress may engage in greater screen use as a coping strategy, while poor sleep quality and prolonged wakefulness may provide greater opportunities for screen use (Alonzo et al., 2021; George et al., 2023; Yang et al., 2020). Therefore, screen time, sleep quality, and psychological well-being may influence one another through complex, interrelated relationships rather than a single causal direction. In China, previous studies have reported significant associations between screen time and sleep quality (Chen et al., 2024), as well as psychological well-being (Cao et al., 2011). High screen time combined with insufficient physical activity has also been associated with a higher prevalence of psychological problems among adolescents (Cao et al., 2011). Likewise, daytime television viewing has been positively associated with insomnia severity among Chinese adolescents (Chen et al., 2024).

Familial factors have also been associated with adolescents’ screen time behaviors. A study in the US found that increased parental monitoring was associated with reduced screen time. It has been suggested that strategies to reduce screen time in adolescence should include improving communication between parents and their children to strengthen family relationships (Al-Shoaibi et al., 2024). According to a study conducted in the Netherlands, adolescents who averaged more than 1 h daily in joint activities with their parents reported lower levels of risk behavior (Koning and Voogt, 2024). Collectively, these studies suggest that parental involvement may be associated with healthier screen time behaviors among adolescents. However, studies on the role of parents in screen time issues in China have not been reported. Chinese parents frequently face stress and demanding work schedules, which often limit their ability to spend time with their children. The study using data from the 2017 China Time Use Survey (CTUS), which explores the relationship between parental time and children’s well-being, found that parental accompaniment is a significant determinant of children’s well-being (Li and Guo, 2023). Therefore, the association between the time parents spend with their children and screen time remains a relatively unexplored area in current research and needs further investigation, particularly within the context of China. Moreover, further research on the role of family socioeconomic status in children’s screen time behavior could offer valuable new insights and deserves closer scrutiny.

The Fujian Adolescent Mental Wellness Study (FAMWeS) is an extensive survey of schoolchildren in Fujian province, focusing on various aspects of adolescent mental health, lifestyle, social issues, and overall well-being. Among the variables assessed in FAMWeS was screen time. Given the growing concerns regarding adolescent screen time, this study examines the prevalence of screen time and investigates its associations with psychological well-being, sleep quality, parental involvement, and family socioeconomic characteristics. Understanding the associations between screen time and psychological well-being, sleep quality, parental involvement, and family socioeconomic status is essential to inform public health strategies and interventions. More importantly, as the largest wellness study conducted to date, FAMWeS comprehensively samples nearly every city in Fujian. Its findings will reveal the levels of screen time, sleep quality, and psychological well-being across these cities, highlighting disparities that can inform future targeted interventions.

Although previous studies and meta-analyses have consistently reported associations between screen time, sleep quality, and psychological distress among adolescents, relatively few have simultaneously examined demographic, family-related, psychological, and sleep-related factors associated with screen time within a single conceptual framework. The present study was guided by the biopsychosocial model, which proposes that health-related behaviors arise from the interaction of biological, psychological, and social factors. In the context of adolescent screen time, demographic characteristics (e.g., age and sex), psychological distress, sleep quality, and family-related characteristics may collectively be associated with screen use behaviors. Furthermore, evidence regarding the associations between screen time and individual domains of sleep quality remains limited. Therefore, this cross-sectional observational study applied PLS-SEM to examine the structural associations among demographic, family-related, psychological, and sleep-related factors and screen time within a proposed conceptual framework. In addition, secondary analyses of individual sleep domains were conducted using the raw summed scores of the corresponding PSQI items to identify which domains were most strongly associated with screen time.

## Methodology

### Sample and setting

The study was conducted in Fujian Province, China, which is situated on the southeast coast of the country. Fujian consists of nine cities: Fuzhou, Xiamen, Zhangzhou, Quanzhou, Sanming, Putian, Nanping, Longyan, and Ningde, along with 9 county-level cities, 31 municipal districts, and 42 counties.

By the end of 2022, Fujian had 1,832 registered secondary schools, comprising 1,273 junior high schools (grades 7–9) and 559 senior high schools (grades 10–12). The total number of registered students was 2,225,397, with 1,526,120 in junior high schools and 699,277 in senior high schools. From May to July 2023, a cross-sectional questionnaire survey on mental health was conducted among adolescent students enrolled in secondary and high schools in Fujian Province using a multi-stage stratified cluster sampling method.

The sampling for the study involved three stages. In the first stage, Fujian Province was divided into three economic development levels based on the 2022 economic development data for all prefecture-level cities published by the Fujian Provincial Bureau of Statistics), and two cities were randomly selected from each level, resulting in a total of seven cities: Fuzhou, Zhangzhou, Ningde, Longyan, Putian, Quanzhou, and Nanping. Economically high-developed regions included Fuzhou City (Eastern Fujian), Xiamen City, Quanzhou City, and Zhangzhou City (Southern Fujian). Medium-developed regions included Ningde City (Eastern Fujian) and Longyan City (Western Fujian). Underdeveloped regions included Putian City (Central Fujian), Nanping City, and Sanming City (Northern Fujian). The survey was carried out in seven prefecture-level cities: Fuzhou City (comprising 1 county-level city, 6 municipal districts, and 6 counties), Zhangzhou City (including 4 municipal districts and 7 counties), Ningde City (with 2 county-level cities, 1 municipal district, and 6 counties), Longyan City (featuring 1 county-level city, 3 municipal districts, and 4 counties), Putian City (consisting of 4 municipal districts and 1 county), Quanzhou City (comprising 4 county-level cities, 4 municipal districts, and 5 counties),and Nanping City (comprising 3 county-level cities, 2 municipal districts, and 5 counties). All administrative regions (cities, districts) within these prefecture-level cities were included in the survey.

In the second stage, a list of secondary and high schools within the selected cities was compiled and categorized. Using simple random sampling, one secondary school and one high school were chosen from each administrative area. In the third stage, cluster sampling was employed to select at least 30% of classes from each grade (1st, 2nd, and 3rd) within the chosen schools, with all students in the selected classes participating in the study.

### Sample size calculation

The sample size is calculated using the formula *n* = design effect (DEFF) u^2^ a/2 p(1-p)/d^2^ (15) with a confidence level of 95% and a corresponding u-value of 1.96. The design effect (DEFF) is set at 1.5, and d is defined as 10% of p. Based on previous studies, the probability (p) of mental health problems is assumed to be 20%. The estimated sample size for each stratum is 7,540. Given there are 6 strata (6 grades), the minimum required sample size is 45,240.

### Data collection

The study employed a systematic approach where teachers in selected classes assisted students in completing paper questionnaires during class time. Initially, the research team collaborated with education administration departments across seven cities, presenting the study’s objectives, significance, and expected outcomes to gain their support. The departments facilitated initial communication and arrangements with the chosen schools. The research team then engaged directly with principals and teachers to explain the study’s background, requirements, and procedures and the necessary school cooperation. Teachers attended two online training sessions to understand the questionnaire content and classroom protocols. Electronic copies of the questionnaires were sent to schools for printing and distribution, with classroom teachers overseeing the distribution, completion, and collection processes. All students in the selected classes received consent forms, and each student returned them, indicating their willingness to participate. There were no refusals or withdrawals, and all selected students completed the questionnaire, which typically took about 20 min. Completed questionnaires subsequently underwent quality-control screening, and questionnaires with inconsistent responses or evidence of straight-lining were excluded before analysis.

To ensure the validity and reliability of responses, the research team has implemented quality control measures, including the use of two trap questions within the questionnaire to detect inconsistent answers. Firstly, responses to Question 1.15 (“Do you have siblings?”) and Question 1.31 (“How many siblings do you have, excluding yourself?”) are reviewed. If a participant answers “No” to Question 1.15 but reports having one or more siblings in Question 1.31, the questionnaire is considered invalid and excluded from analysis. Secondly, responses to Question 1.2 (“Where do you come from, current place of residence?”) and Question 1.45 (“What is your current place of residence?”) are evaluated. Although both questions ask about rural, urban, and county residences, inconsistencies in the order of responses indicate discrepancies. Consequently, surveys with conflicting answers to these questions are also excluded to maintain the dataset’s integrity. Additionally, responses exhibiting straight-lining, where identical answers are provided throughout, are removed.

### Instrument

The FAMWeS represents a comprehensive study of schoolchildren in Fujian province, exploring various aspects of adolescent mental health, lifestyles, family dynamics, and overall well-being. The survey questionnaire used in FAMWeS is available in Supplementary Appendix 1. This paper uses data from the FAMWeS, with variables such as demographics, emotional state, and psychological distress considered as independent factors. Emotional state was assessed using the Depression, Anxiety, and Stress Scale for Youth (DASS-Y), and sleep quality was measured using the Pittsburgh Sleep Quality Index (PSQI). Demographic data encompassing age, gender, location, academic performance, maternal and paternal education levels, and time spent with parents were included.

#### Depression, anxiety, and stress scale for youth (DASS-Y)

Emotional state was evaluated using Depression, Anxiety, and Stress Scale for Youth (DASS-Y) (Szabo and Lovibond, 2022). The DASS-Y is a version of the Depression, Anxiety, and Stress Scale for Youth – 21 items (DASS-21) for youth aged 7–18 years of age designed to measure the negative emotional states of depression, anxiety and stress (Szabo and Lovibond, 2022). The cutoff scores for the DASS-Y were determined using the same percentile ranges as those for the adult DASS. These cutoffs classify scores into levels of normal, mild or moderate, and severe or extremely severe for depression, anxiety, and stress (Szabo and Lovibond, 2022). The Chinese version of the DASS-Y was utilized, and it showed superior robustness compared to the DASS-21 in terms of psychometric properties (Cao et al., 2023).

#### Pittsburgh sleep quality index (PSQI)

Sleep quality was assessed with the Pittsburgh Sleep Quality Index (PSQI) (Buysse et al., 1989). The scale consists of 19 items and covers seven components including “subjective sleep quality,” “sleep latency,” “sleep duration,” “habitual sleep efficiency,” “sleep disturbances,” “use of sleep medication,” and daytime dysfunction. The sum of seven components is called the PSQI global score. Each component is scored from 0 to 3, resulting in a global score ranging from 0 to 21, with lower scores indicating better sleep quality. Participants with a PSQI global score of 0–5 were classified as having good sleep quality, whereas those with a score greater than 5 were classified as having poor sleep quality, consistent with the original PSQI scoring criteria (Buysse et al., 1989). The PSQI is one of the most rigorously validated questionnaires to assess sleep quality. The PSQI has demonstrated excellent diagnostic validity, with a sensitivity of 89.6% and specificity of 86.5% using the recommended cut-off of **>** 5 for poor sleep quality (Buysse et al., 2008). Since Chinese is the official language in China and most students are not proficient in English, the Chinese version of the Pittsburgh Sleep Quality Index (Buysse et al., 1989) was utilized in this study (Buysse et al., 1989; Lu et al., 2014). The Chinese PSQI is a commonly used self-reported scale to measure sleep quality in adolescents in many studies conducted in China and showed good reliability (Cronbach coefficient = 0.845) and good validity (Lu et al., 2014). In the present study, the Cronbach’s alpha for the PSQI was 0.707, indicating acceptable internal consistency.

#### Screen time

The questionnaire on screen time asked participants, “How many hours per day do you use electronic devices?” The measure focused on overall screen media usage, capturing the total duration or frequency of use across various platforms, including computers, the internet, mobile phones, television, and video games. The response options were: “< 30 min,” “30 min to 1 h,” “1 to 4 h,” and “> 4 h.” Additionally, participants were asked to select the reasons for their electronic device use from multiple-choice options such as studying, looking up information, watching videos for entertainment, and playing games.

### Statistical analyses

The sample characteristics were described using mean ± standard deviation (SD) or frequency based on the type of variables. Cronbach’s alpha coefficient was also determined to evaluate the internal consistency of DASS-Y. In this study, Cronbach’s α for three subscales of DASS-Y were 0.893 for depression, 0.861 for anxiety, and 0.868 for stress. The scale demonstrated good reliability, with an alpha coefficient between 0.6 and 0.7 indicating an acceptable level of reliability, and 0.8 or greater indicating a very good level (Hulin et al., 2001). Multivariable logistic regression analyses were then performed to assess the associations between screen time and the individual sleep domains. Partial least squares structural equation modeling (PLS-SEM) was applied to examine the structural associations among the study variables within the proposed conceptual framework and to identify factors associated with screen time. PLS-SEM was selected because the objective of this study was to explore and estimate the relative associations of multiple correlated explanatory variables with screen time rather than to test or confirm a well-established theoretical model. All demographic, family-related, psychological, and sleep-related variables were entered simultaneously into the PLS-SEM model. Consequently, each standardized path coefficient represents the association between the explanatory variable and screen time after adjustment for the other variables included in the model. Compared with conventional multiple regression, PLS-SEM enables the simultaneous estimation of multiple relationships while accounting for correlations among the explanatory variables and provides standardized path coefficients for comparing the relative strength of these associations. Covariance-based SEM was not considered necessary because the study did not aim to evaluate overall model fit or confirm an established latent variable structure. Bootstrapping with 5,000 resamples was performed to estimate the significance of the path coefficients using SmartPLS version 3.2.8 (SmartPLS GmbH). Given the cross-sectional design, the estimated path coefficients represent statistical associations rather than causal relationships. The discriminant validity using Heterotrait-Monotrait (HTMT) < 0.9, the model fit index evaluation: Standardized Root Mean Square Residual (SRMR) < 0.08, Normed Fit Index (NFI) > 0.90, the effect Size (f^2^) and predictive relevance (Q^2^ > 0) were assessed (Hair et al., 2021; Hair et al., 2019).

Screen time was specified as the dependent (outcome) variable, whereas demographic characteristics (e.g., age and sex), family-related factors (e.g., parental education and time spent with parents), psychological distress (depression, anxiety, and stress), and sleep quality were specified as explanatory (independent) variables to identify factors associated with screen time. For the domain-specific analyses of sleep quality, the raw summed scores of the corresponding PSQI items were used rather than the standardized PSQI component scores (0–3). This approach retained greater variability in the domain-specific outcome measures. The primary objective of the study was to examine associations rather than estimate population prevalence; therefore, sampling weights were not applied. The multistage stratified cluster sampling design was incorporated during sample size determination through the use of a design effect, although the subsequent regression analyses treated observations as independent. No mediation analysis was performed. Given the cross-sectional design, the estimated path coefficients represent statistical associations and should not be interpreted as causal relationships. This analytic approach enables the estimation of standardized path coefficients and their statistical significance while accounting for correlations among the explanatory variables. Of note, categorical and ordinal variables were numerically coded before analysis. Gender was coded as male = 0 and female = 1. Study grade was coded in ascending order from Grade 7 = 1 to Grade 12 = 6, with higher values representing higher grade levels. Academic performance was coded in ascending order according to performance ranking, with higher values indicating better academic performance. Paternal and maternal education were coded in ascending order of educational attainment, with higher values representing higher education levels. Time spent with fathers and mothers was coded in ascending order of duration, with higher values indicating more time spent with the respective parent. Age, PSQI global score, and the DASS-Y depression, anxiety, and stress scores were entered as continuous variables.

## Results

A total of 58,381 questionnaires were collected, of which 48,432 complete responses were included in the final analyses reported in this paper. As shown in Table 1, the age distribution of participants showed that the majority were aged 12–14 years (39.8%) and 15–17 years (56.9%). There was an almost equal proportion of males (50.6%) and females (49.4%). The majority of participants reported that the highest paternal education level was high school or college (36.2%), and similarly, the highest maternal education level was high school or college (37.6%). In terms of academic achievement, 29.8% of participants reported being in the 30–50% range of their class (Grade C), followed by 23.7% who reported being in the top 15–30% (Grade B), and only 16.7% reported being in the top 15% (Grade A).

**TABLE 1:** Descriptive statistics for participants’ demographic and measurement information (*N* = 48432).

Factors	*N* (%)
**Socio-demographic characteristics**	
Age group (years)	
12–14	19261 (39.8)
15–17	27580 (56.9)
18–19	1591 (3.3)
Gender	
Male	24492 (50.6)
Female	23940 (49.4)
Study grade	
Top 15% (A level)	8107 (16.7)
15–30% (B level)	11497 (23.7)
30–50% (C level)	14425 (29.8)
50–75% (D level)	10049 (20.7)
> 75% (E level)	4354 (9.0)
Father’s highest education	
Elementary school	6694 (13.8)
Junior high school	9724 (20.1)
High school/ Junior college	17544 (36.2)
Post-secondary	8396 (17.3)
Undergraduate	2938 (6.1)
Postgraduate	3136 (6.5)
Mother’s highest education	
Elementary school	6598 (13.6)
Junior high school	7145 (14.8)
High school/ Junior college	18222 (37.6)
Post-secondary	9271 (19.1)
Undergraduate	3163 (6.5)
Postgraduate	4033 (8.3)
**Time spent with parents**	
Father	
Unaccompanied	8495 (17.5)
Less than 1 h/day	13996 (28.9)
1 to 2 h/day	9456 (19.5)
2 to 3 h/day	5486 (11.3)
More than 3 h/day	10999 (22.7)
Mother	
Unaccompanied	2998 (6.2)
Less than 1 h/day	8055 (16.6)
1 to 2 h/day	8039 (16.6)
2 to 3 h/day	7151 (14.8)
More than 3 h/day	22189 (45.8)
**DASS-Y**	
Depression	
Normal	39115 (80.8)
Mild/ Moderate	7562 (15.6)
Severe/ Extremely severe	1755 (3.6)
Anxiety	
Normal	37641 (77.7)
Mild/ Moderate	9145 (18.9)
Severe/ Extremely severe	1646 (3.4)
Stress	
Normal	43879 (90.6)
Mild/ Moderate	3382 (7.0)
Severe/ Extremely severe	1171 (2.4)
**Sleep quality**	
PSQI	
0–5	23165 (47.8)
6–21	25267 (52.2)

**FIGURE 1:**
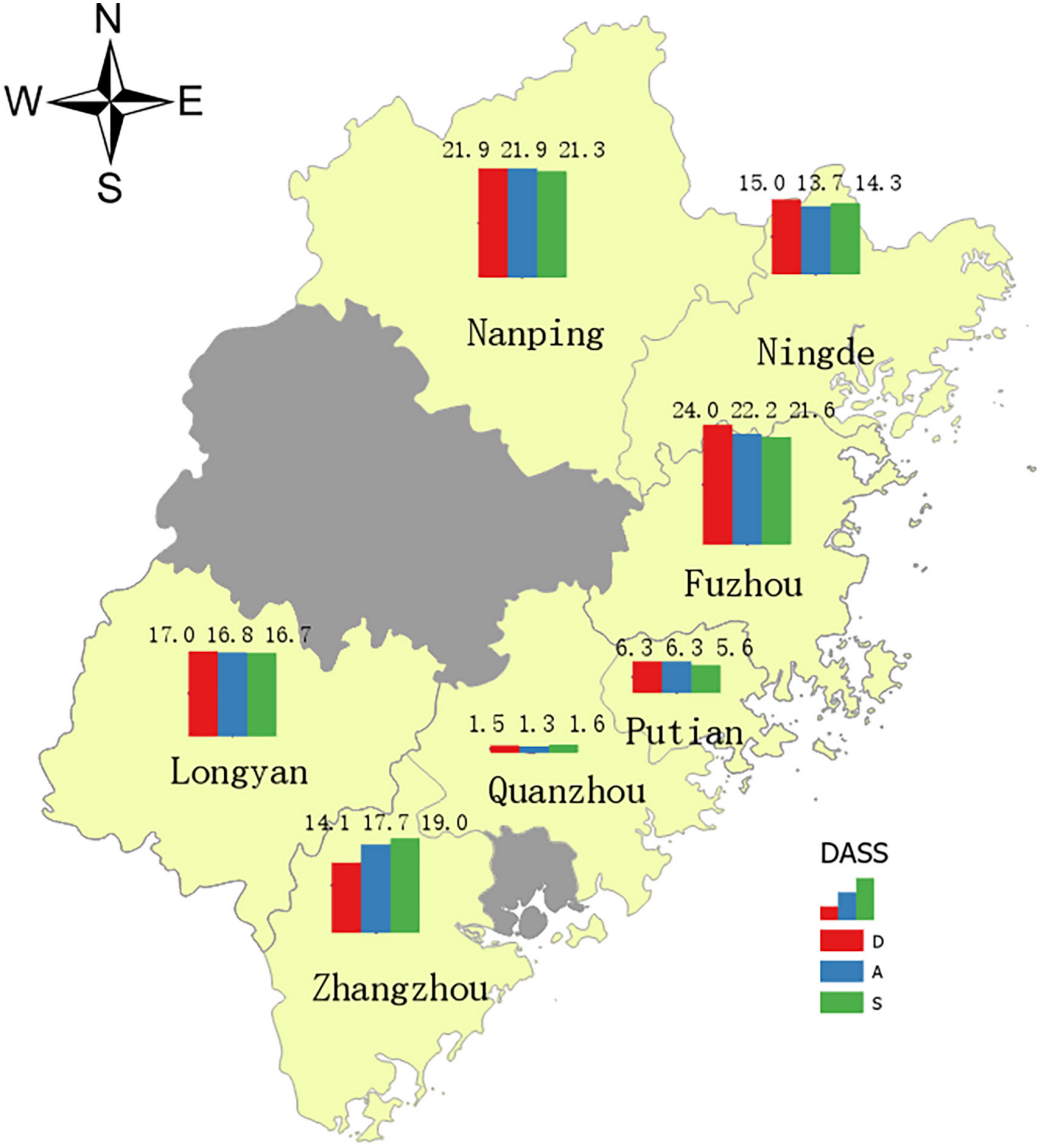
Proportion of depression, anxiety, and stress across different cities in Fujian province.

The descriptive results of the DASS-Y for all participants are presented in the first and second columns of Table 1. Overall, the majority of respondents exhibited normal levels of depression (80.8%), anxiety (77.7%), and stress (90.6%). However, a notable minority experienced mild to moderate or severe to extremely severe levels of depressive symptoms (15.6%), anxiety (18.9%), and stress (7.0%). Figure 1 displays the distribution of depression, anxiety, and stress across different cities. Fuzhou reported the highest proportions, with depression at 24.0%, anxiety at 22.2%, and stress at 21.6%. Nanping followed with depression and anxiety both at 21.9%, and stress at 21.3%. Putian had lower rates, with depression and anxiety both at 6.3%, and stress at 5.6%, while Quanzhou recorded the lowest levels, with depression at 1.5%, anxiety at 1.3%, and stress at 1.6%.

### PSQI

The overall mean global PSQI score was 5.88 ± 3.17, with a range of 0 to 21. A total of 52.2% of overall participants had scores > 5, indicating poor sleep quality, while 47.8% had scores from 0 to 5. Supplementary Figure 1 shows the proportion of participants with poor sleep quality (PSQI scores ≥ 5) across various cities in Fujian Province. The highest proportion of poor sleep quality was recorded in Fuzhou (21.8%), followed by Zhangzhou (19.7%) and Nanping (19.1%). The lowest proportion of poor sleep quality was in Quanzhou (1.8%).

Table 2 presents the findings of the PSQI components, and multivariable logistic regression analyses of the associations of components of poor sleep quality with screen time. A total of 26.8% reported their subjective sleep quality as fairly bad or very bad. The odds of having very bad subjective sleep quality were significantly 1.57 times higher (OR 1.57, 95% CI 1.36–1.81) than those with very good subjective sleep quality in those with screen time greater than 4 h compared to those with very good subjective sleep quality and 4 h or less of screen time. For sleep latency, 16.1% reported taking between 31–60 min to fall asleep, while 9.5% reported taking more than 60 min. There were significantly higher odds of having a sleep latency of 31–60 min (OR 1.15, 95% CI 1.04–1.27) compared to those with a sleep latency of less than 15 min among those who have screen time greater than 4 h compared to those with 4 h or less.

**TABLE 2:** Multivariate analysis associations between Pittsburgh Sleep Quality Index (PSQI) components and participants’ screen time (*N* = 48432).

Pittsburgh Sleep Quality Index (PSQI)		Screen time			Screen time
Sleep quality component	*N* (%)	≤ 4 h (*n* = 42971)	> 4 h (*n* = 5461)	*p*-value	> 4 h vs. ≤ 4 h
					aOR (95% CI)
Subjective sleep quality					
Very good	11879 (24.5)	10796 (90.9)	1083 (9.1)	*p* < 0.001	Reference
Fairly good	23595 (48.7)	21336 (90.4)	2259 (9.6)		0.95 (0.87–10.3)
Fairly bad	10486 (21.7)	8982 (85.7)	1504 (14.3)		1.11 (0.99–1.23)
Very bad	2472 (5.1)	1857 (75.1)	615 (24.9)		1.57 (1.36–1.81)***
Sleep latency					
< 15 min	15481 (32.0)	14039 (90.7)	1442 (9.3)	*p* < 0.001	Reference
16–30 min	20555 (42.4)	18511 (90.1)	2044 (9.9)		0.99 (0.91–1.07)
31–60 min	7806 (16.1)	6680 (85.6)	1126 (14.4)		1.15 (1.04–1.27)**
> 60 min	4590 (9.5)	3741 (81.5)	849 (18.5)		1.12 (0.99–1.26)
Sleep duration					
> 7 h	10844 (22.4)	9903 (91.3)	941 (8.7)	*p* < 0.001	Reference
6–7 h	22632 (46.7)	20498 (90.6)	2134 (9.4)		1.01 (0.93–1.09)
5–6 h	12172 (25.1)	10444 (85.8)	1728 (14.2)		1.29 (1.18–1.42)***
< 5 h	2784 (5.7)	2126 (76.4)	658 (23.6)		1.51 (1.30–1.74)***
Habitual sleep efficiency					
> 85%	44703 (92.3)	40010 (89.5)	4693 (10.5)	*p* < 0.001	Reference
75–84%	1997 (4.1)	1596 (79.9)	401 (20.1)		1.48 (1.29–1.70)***
65–74%	819 (1.7)	677 (82.7)	142 (17.3)		1.44 (1.18–1.75)***
< 65%	913 (1.9)	688 (75.4)	225 (24.6)		2.02 (1.69–2.42***
Sleep disturbances					
0	9523 (19.7)	8694 (91.3)	829 (8.7)	*p* < 0.001	Reference
1–9	33347 (68.9)	29765 (89.3)	3582 (10.7)		1.09 (0.99–1.19)
10–18	4904 (10.1)	4039 (82.4)	865 (17.6)		1.30 (1.14–1.47)***
19–27	658 (1.4)	473 (71.9)	185 (28.1)		1.51 (1.22–1.89)***
Use of sleep medication					
No	46189 (95.4)	41039 (88.9)	5150 (11.1)	*p* < 0.001	Reference
Less than once	1285 (2.7)	1170 (91.1)	115 (8.9)		0.72 (0.59–0.88)**
Once or twice	478 (1.0)	407 (85.1)	71 (14.9)		0.96 (0.74–1.25)
Three or more times	480 (1.0)	355 (74.0)	125 (26.0)		1.19 (0.95–1.50)
Daytime dysfunction					
0	10172 (21.0)	9283 (91.3)	889 (8.7)	*p* < 0.001	Reference
1–2	12256 (25.3)	11253 (91.8)	1003 (8.2)		0.92 (0.83–1.02)
3–4	17825 (36.8)	15755 (88.4)	2070 (11.6)		1.20 (1.09–1.33)***
5–6	8179 (16.9)	6680 (81.7)	1499 (18.3)		1.56 (1.40–1.75)***

**p < 0.01,

***p < 0.001.

Regarding sleep duration, 46.7% reported sleeping 6–7 h, with a considerable proportion sleeping 5–6 h (25.1%). There were significantly higher odds of having a sleep duration of less than 5 h (OR 1.51, 95% CI 1.30–1.74) and of having a duration of 5–6 h (OR 1.29, 95% CI 1.18–1.42) compared to the reference duration of more than 7 h in those with screen time greater than 4 h compared to those with screen time of 4 h or less. In terms of habitual sleep efficiency, a high proportion (92.3%) reported an efficiency of more than 85%. There were significantly higher odds (OR 2.02, 95% CI 1.69–2.42) of having the lowest efficiency ( < 65%) compared to the highest efficiency (> 85%) in those with screen time greater than 4 h.

The majority (68.9%) reported a sleep disturbance score of 1–9, and a large proportion reported not using any sleep medication (95.4%). Those with screen time exceeding 4 h had significantly higher odds of scoring between 19 and 27 for sleep disturbances compared to the reference group with no sleep disturbances (OR 1.51, 95% CI 1.22–1.89). The majority (36.8%) reported a daytime dysfunction score of 3–4, while 16.9% reported a high daytime dysfunction score of 5–6. There were significantly higher odds of having a daytime dysfunction score of 5–6 (OR 1.56, 95% CI 1.40–1.75) and a score of 3–4 (OR 1.20, 95% CI 1.09–1.33) compared to the reference group with no daytime dysfunction in those with screen time greater than 4 h.

### Screen time

**FIGURE 2:**
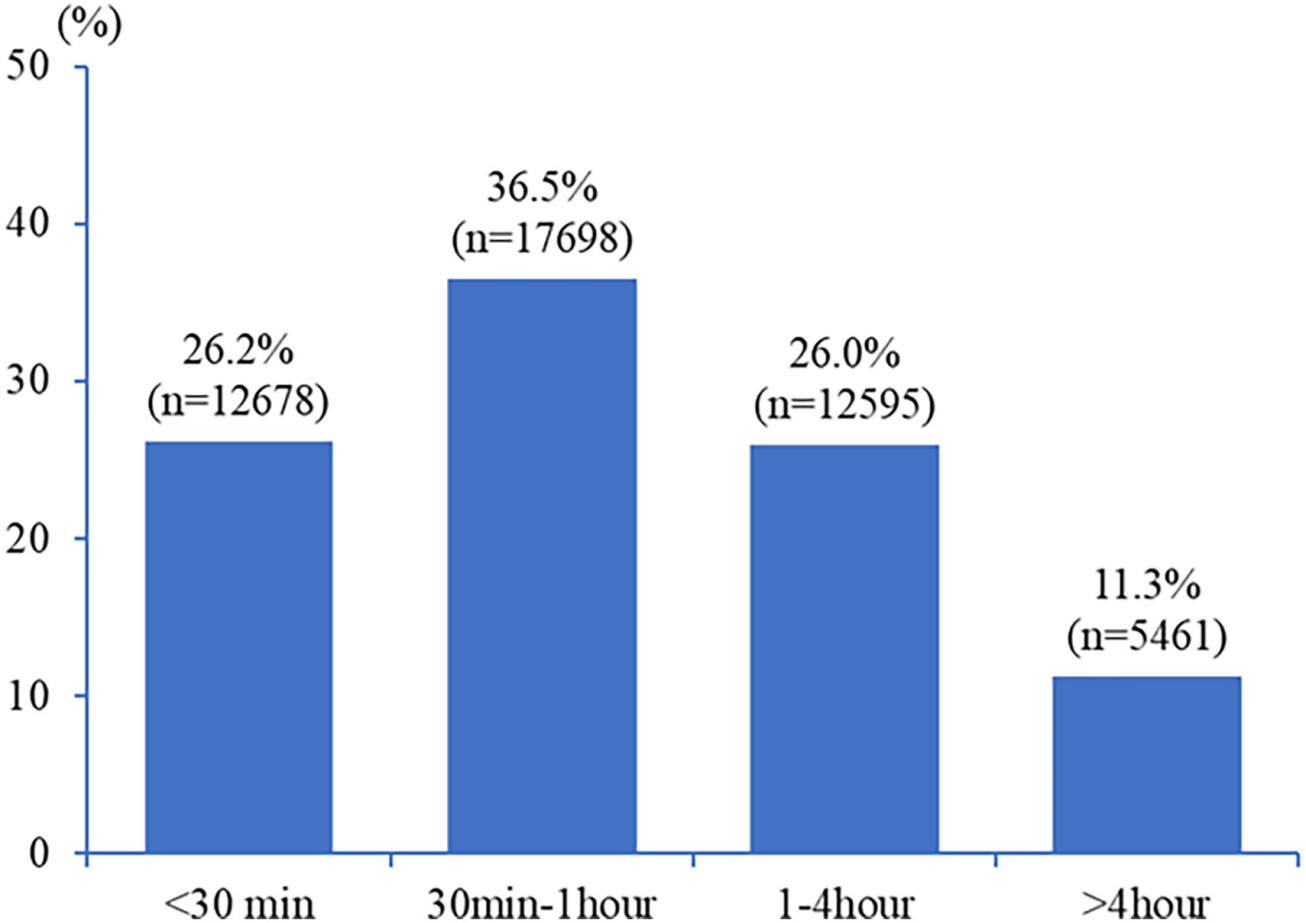
Overall screen time per day.

Figure 2 shows that the largest group, 36.5%, reported a screen time of 30 min to 1 h per day. This was followed by 26.2% who reported less than 30 min per day and 26.0% who reported 1 to 4 h per day. Meanwhile, 11.3% reported more than 4 h per day. The descriptive findings on the usage of electronic devices revealed that 52.6% of participants used them for studying, 56.3% for looking up information, 59.4% for video entertainment, and 50.9% for playing games. Supplementary Figure 2 illustrates the proportion of participants with more than 4 h of daily screen time across various cities in Fujian Province. Zhangzhou had the highest proportion, with 27.2% of participants reporting over 4 h per day, followed by Nanping at 20.5% and Fuzhou at 18.8%. Quanzhou reported the lowest proportion at 1.8%, while Ningde and Putian recorded 9.1% and 10.7%, respectively.

**FIGURE 3:**
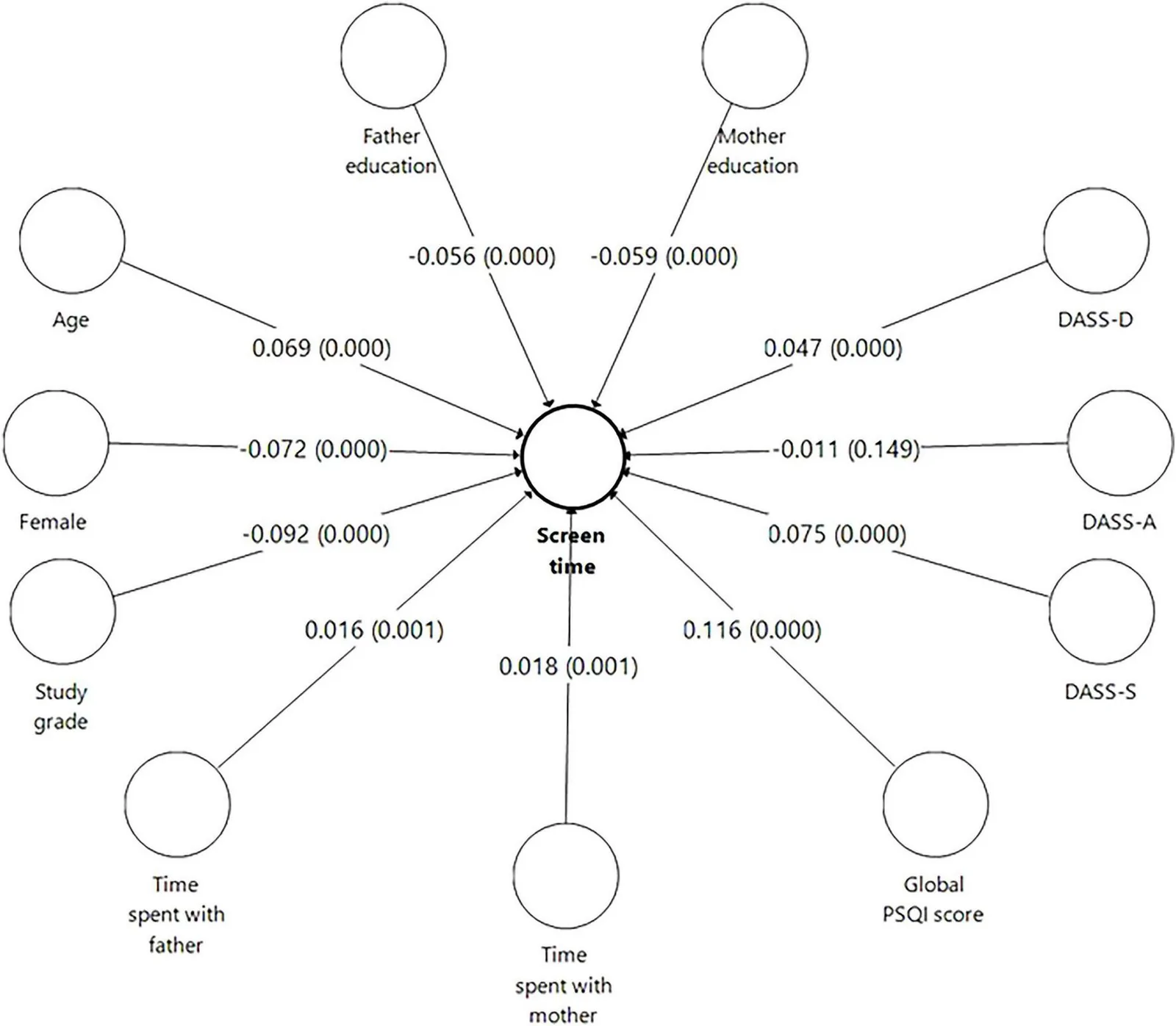
PLS-SEM model showing factors associated with screen time.

Figure 3 illustrates the PLS path model of factors associated with screen time. Higher screen time was significantly linked to older age (β = 0.069, *p* < 0.001) and being male (β = −0.072, *p* < 0.001). Study grade showed a significant inverse relationship with screen time (β = −0.092, *p* < 0.001). Both paternal (β = −0.056, *p* < 0.001) and maternal (β = −0.059, *p* < 0.001) education were significantly inversely associated with screen time. Time spent with fathers (β = 0.016, *p* = 0.001) and mothers (β = 0.018, *p* = 0.001) was significantly positively associated with screen time. The PSQI global score was positively associated with screen time, with the highest path coefficient (β = 0.116, *p* < 0.001). Depression (β = 0.047, *p* < 0.001) and stress (β = 0.075, *p* < 0.001) scales of DASS-Y were also significantly positively associated with screen time. The adjusted R^2^ was 0.069, indicating that the variables included in the model explained 6.9% of the variance in screen time, suggesting that additional factors not examined in this study may also contribute to adolescents’ screen time behavior. Discriminant validity was established as all Heterotrait-Monotrait (HTMT) correlation ratios were below the recommended threshold of 0.90. Regarding effect sizes, all predictive variables yielded an *f*^2^ value below 0.02, indicating no substantive effect on screen time. The model exhibited a small predictive relevance for screen time, evidenced by a *Q*^2^ value of 0.069. Finally, model fit indices indicated a perfect fit to the data, with a Standardized Root Mean Square Residual (SRMR) = 0.000 and a Normed Fit Index (NFI) = 1.000. These specific fit values are expected given the single-endogenous structural design of the model, which yields zero degrees of freedom.

## Discussion

This study examined adolescent screen time, psychological well-being, and sleep quality using data from FAMWeS, highlighting disparities across different cities. The findings reveal an intriguing interplay between screen time, psychological well-being, sleep quality, parental involvement, and various individual and family factors.

The descriptive results from the DASS-Y reveal a complex picture of adolescent mental health across different cities in Fujian Province. While the majority of respondents displayed normal levels of depression, anxiety, and stress, there remains a significant minority experiencing varying degrees of psychological distress. Specifically, 15.6% reported mild to severe depressive symptoms, 18.9% reported anxiety, and 7.0% experienced stress. When comparing our findings to a recent large-scale study conducted among adolescents in Jiangsu Province using the DASS-21, which reported that 14.6% exhibited depressive symptoms, 8.0% showed evidence of anxiety, and 27.4% demonstrated signs of stress (Wang H. et al., 2023), it appears that adolescents in Fujian have relatively higher levels of anxiety but lower levels of stress.

The geographic distribution of these mental health issues is particularly revealing. Fuzhou stands out with the highest proportions of depression (24.0%), anxiety (22.2%), and stress (21.6%), indicating that adolescents in this city may be experiencing more pronounced psychological distress compared to their peers in other regions. Nanping follows closely, with similarly elevated levels of depression and anxiety (both at 21.9%) and stress (21.3%). It appears that economic development does not have a straightforward impact on adolescent mental health. While Fuzhou, a highly developed region, shows the highest levels of depression, anxiety, and stress. Nanping, a relatively underdeveloped area, also exhibits similarly elevated levels of psychological distress. In contrast, Quanzhou, another developed city, recorded the lowest levels of depression, anxiety, and stress. This suggests that other factors beyond economic development may be contributing to these mental health challenges. Studies have indicated that variations in social support systems, educational pressures, cultural influences, school environments, environmental stressors, or individual resilience could significantly influence stress (McMahon et al., 2013; Collins et al., 2024) with these factors potentially varying uniquely across each city in Fujian Province. These findings underscore the need for a deeper investigation into the local factors influencing adolescent mental health in different regions of China. Furthermore, caution is warranted when generalizing these findings beyond Fujian Province. Cultural norms, educational systems, socioeconomic conditions, patterns of digital technology use, and access to electronic devices vary considerably across different regions of China and between countries. These contextual differences may influence adolescents’ screen-use behaviors, sleep quality, and psychological well-being, and therefore the observed associations may not be directly applicable to other populations. Future multicenter studies involving more diverse geographic regions and international populations are needed to evaluate the consistency and generalizability of these findings.

The overall mean global PSQI score of 5.88 indicates a high level of poor sleep quality among adolescents in Fujian Province, and further with 52.2% scoring over 5, indicating that over half may face sleep-related issues that potentially impact their health and well-being. When comparing the distribution of poor sleep quality across different cities, Fuzhou stands out with the highest proportion at 21.8%, followed by Zhangzhou (19.7%) and Nanping (19.1%). Both Fuzhou and Zhangzhou are economically developed, whereas Nanping is less economically developed. Interestingly, Quanzhou, another economically developed city, recorded the lowest proportion of poor sleep quality at just 1.8%. Similarly, the findings perhaps imply that factors beyond economic development, such as cultural practices, lifestyle differences, or local environmental conditions, may play a significant role in influencing sleep quality among adolescents in different cities. These findings highlight the need for further research to understand the specific factors contributing to these regional differences in sleep quality.

The current study found that 26.0% of students used screens for 1 to 4 h daily, with 11.3% exceeding 4 h, suggesting potentially excessive screen use. Findings from two nationally representative Health Behavior in School-aged Children (HBSC) surveys conducted in 2010 and 2014 across 44 European and North American countries show that adolescents who reported watching TV for more than 4 h per day had higher odds of experiencing psychosomatic complaints compared to those who watched 2 h or less per day (Khan et al., 2022). The China 2018 Report Card on physical activity for children and youth reported that only 13.1% of students were physically active for 60 min daily, while 92.9% had 2 h or more of screen time per day (Liu et al., 2019). However, a direct comparison with the current study is challenging, as the current study did not employ the 2-h screen time cutoff.

Screen time disparities by city reveal that Zhangzhou has the highest proportion of participants reporting over 4 h of daily screen time, followed by Nanping and Fuzhou. Interestingly, while Zhangzhou and Fuzhou are economically developed cities, Nanping is relatively underdeveloped. Quanzhou, another developed city, reported the lowest proportion of screen time. This suggests that there is no consistent correlation between economic development and screen time in this study. However, according to the 2019 Children’s Blue Book: China Children’s Development Report, children in rural towns spend significantly more time using electronic devices (108.18 min) compared to their urban counterparts (88.40 min) (Yuan et al., 2019).

The PLS path model reveals several significant factors associated with screen time among adolescents. In this study, male gender was positively associated with higher screen time in adolescents, with screen time predominantly higher in males. This finding is consistent with a study conducted in Sweden, where it was also observed that males spent more time on screens compared to females (Dahlgren et al., 2021). This gender difference in screen time could be attributed to several factors. Boys tend to be more involved in screen-intensive activities like video gaming, as noted in other studies (Oswald et al., 2020). Additionally, boys often enjoy watching sports and exploring technology, which may also partly explain their higher screen time. The study also found that screen time increased with age, a trend similarly observed in other research. The possible reason why increasing age is positively associated with higher screen time in adolescents is that, as they grow older, they gain more autonomy and access to personal devices, and tend to spend more time using screens for social interaction, entertainment, and academics.

Additionally, higher study grade was inversely associated with screen time. However, given the positive association between age and screen time and the cross-sectional design, this finding should be interpreted cautiously. A meta-analysis found no association between overall screen media use and academic performance, but television viewing and video game playing were inversely related to composite academic performance scores (Adelantado-Renau et al., 2019). Similarly, another meta-analysis study revealed a lack of association between overall screen media use and academic performance, however, when the association between each screen-based activity and academic performance was analyzed, television viewing and video gaming were inversely associated with academic performance, suggesting these activities may have the most negative impact on academic performance (Adelantado-Renau et al., 2019). It is important to note that the screen time measure combined both academic and recreational screen use into a single exposure. Although participants reported their reasons for using electronic devices, the duration devoted to each activity was not recorded. Consequently, adolescents with the same total screen time may have had substantially different patterns of screen use. This may have attenuated or obscured activity-specific associations because recreational screen use has generally been reported to show stronger associations with poor sleep quality and psychological distress than educational screen use. Therefore, the associations observed in this study should be interpreted as reflecting overall screen exposure rather than the effects of any specific type of screen-based activity.

It is also important to recognize that not all screen time should be regarded as detrimental. Electronic devices have become an integral component of modern education, and a substantial proportion of adolescents’ daily screen use is devoted to schoolwork, learning, and information seeking. Consequently, the findings should not be interpreted as indicating that all increases in screen time have negative health implications. Instead, they suggest that overall screen exposure is associated with sleep quality and psychological well-being, while the specific contribution of educational and recreational screen use could not be distinguished in the present study. Public health recommendations should therefore focus on promoting healthy and balanced screen-use behaviors, particularly by discouraging excessive recreational and nighttime screen use, rather than indiscriminately reducing total screen time.

Furthermore, the descriptive findings on the usage of electronic devices revealed that participants used electronic devices almost equally for both recreational and non-recreational purposes. Therefore, in this study, it is difficult to conclude whether the association between screen time and academic performance is primarily related to recreational or non-recreational use. Future studies should distinguish between these forms of screen use, as they may have different associations with adolescent mental health and sleep quality.

In this study, the PLS-SEM model demonstrated significant inverse associations between both paternal and maternal education levels and screen time. This suggests that higher parental education levels may be associated with better regulation or awareness of adolescents’ screen use. Similarly, data from the European Youth Heart Study identified parental education as the most significant socioeconomic factor influencing screen-based behaviors in children (De Craemer et al., 2018). A potential explanation for the inverse relationship between parental education and screen time is that lower parental education is associated with reduced parental modeling, such as setting a good example (Gebremariam et al., 2015). Additionally, research shows that children’s screen time is influenced by parenting practices, such as establishing rules to positively guide behavior, which tend to be less common in families with lower socioeconomic status (Ventura and Birch, 2008). Therefore, it is important to support parents with lower levels of education in managing their children’s screen time. Interestingly, time spent with both fathers and mothers was positively associated with screen time, suggesting that family interactions, while valuable, may also involve screen-based activities. This could be due to shared activities such as watching screens together. However, it was reported that families who watch TV together every day have a 1.84-times higher chance of excessive TV watching. Therefore, parents should be mindful of screen time habits and strive to set limits on daily TV watching to promote healthier viewing practices (Brindova et al., 2014). Although age, sex, and parental education were statistically associated with screen time, the standardized effect sizes were small, suggesting that these factors contribute only modestly to variations in adolescents’ screen time. Although these associations reached statistical significance, their practical significance is likely to be modest, particularly given the large sample size of the present study, which provides substantial statistical power to detect relatively small effect sizes. Beyond parental education, broader socioeconomic and lifestyle factors may also be associated with adolescents’ screen use, sleep quality, and psychological well-being. Previous studies have suggested that socioeconomic status and physical activity may jointly relate to these outcomes, highlighting the importance of considering these contextual factors in future research (Rassolnia and Nobari, 2024). Future studies should consider incorporating additional socioeconomic indicators and lifestyle behaviors, such as physical activity, to better understand their interplay with screen time.

The partial least squares structural equation modeling (PLS-SEM) also demonstrated a positive association between depression and stress scales from the DASS-Y and screen time. The link between screen time and mental health is well-established in previous studies, and our study similarly confirms this association in the adolescent population of Fujian Province. Meta-analysis findings have shown that screen time, encompassing daily use of digital technology across various devices including social media, is associated with increased risks of depression, anxiety, and loneliness in adults (Santos et al., 2023). Another meta-analysis highlighted that screen time is associated with depressive symptoms across various age groups, including children, adolescents, and the elderly (Li et al., 2022).

Regarding the severity of mental health issues, the current study found that 15.6% of participants reported mild to severe depressive symptoms, 18.9% experienced anxiety, and 7.0% reported stress, which is consistent with previous findings. A recent large-scale study in Jiangsu province found that the prevalence of mental health disorders among children and youth was 17.5%, a figure that has been steadily increasing in recent years (Wang H. et al., 2023). In response to rising concerns about child and adolescent mental health in China, the Chinese Health Commission, Ministry of Education, and other government departments initiated a joint action plan. By 2022, the plan requires schools to establish psychological service platforms and mandates that 60% of higher-level mental health hospitals offer outpatient services for children and adolescents. Additionally, it calls for the development of psychological assistance hotlines and efforts to raise mental health awareness, aiming to reach 80% of the child and adolescent population (Wang et al., 2020). The findings of this study underscore the need for increased support for mental health services and the utilization of established psychological resources. In addition, psychosocial factors such as loneliness and social anxiety have been linked to problematic screen-related behaviors among adolescents with internet gaming dependence (Norouzian et al., 2024). Although these variables were not assessed in the present study, future research should investigate their potential roles in relation to general screen time behavior.

The finding that sleep quality was positively associated with screen time and had the largest path coefficient among the variables included in the PLS-SEM suggests that sleep quality may be relatively more strongly associated with screen time than the other variables examined. Nevertheless, the standardized path coefficient remained relatively small, indicating that the strength of this association was modest despite its high statistical significance. However, the overall explanatory power of the model was modest, indicating that additional factors not included in the model are also likely to contribute to adolescents’ screen time behavior. Several biological and psychological mechanisms may explain the observed associations between screen time, poor sleep quality, and psychological distress. An umbrella review suggests that evening screen use may delay bedtime, reduce sleep duration, and impair sleep quality through multiple pathways, including circadian disruption associated with light exposure, increased cognitive and physiological arousal, and prolonged engagement with emotionally stimulating digital content (Fiore et al., 2025). Nonetheless, a recent systematic review of studies examining the relationship between youth screen media use and sleep similarly found a significant impact of screen habits on sleep (Hale and Guan, 2015). Suggested mechanisms for this impact include reduced time available for sleep, psychological stimulation, light exposure from electronic devices, and heightened physiological alertness (Hale and Guan, 2015). In addition to the systematic review, many studies similarly highlight the negative effects of screen time on sleep duration and timing in many populations globally. These studies encompass studies on electronic devices used in infants in Thailand, preschool-aged children in the Netherlands (Sijtsma et al., 2015), school-aged children in Canada (Chaput et al., 2014), and adolescents in Saudi Arabia (Al-Hazzaa et al., 2014) and Norway (Hysing et al., 2015). Taken together, these findings underscore the importance of addressing screen time as an important factor in interventions aimed at improving sleep quality among adolescents. However, given the modest explanatory power of the model and the potential influence of unmeasured confounding factors, including physical activity, obesity, chronotype, caffeine consumption, family functioning, and other lifestyle and clinical characteristics, the observed associations should be interpreted cautiously. Screen time should therefore be considered as one component within a broader biopsychosocial framework influencing adolescent sleep quality and psychological well-being, rather than the sole or primary determinant of these outcomes. Therefore, parents should be informed about healthy screen-use habits and encouraged to support balanced screen use alongside other healthy lifestyle behaviors that contribute to adolescent sleep and psychological well-being. Lastly, further investigation into the components of sleep quality revealed that habitual sleep efficiency differed most markedly among adolescents reporting more than 4 h of daily screen time. Habitual sleep efficiency, which measures the proportion of time spent in bed that is spent sleeping, indicates that excessive screen time leads to adolescents having less ideal sleep duration. Parents should be advised to monitor their children’s sleep patterns, even after they have gone to bed, in order to detect any issues with poor habitual sleep efficiency and address them promptly.

## Limitation

While this study has several strengths, including a large sample size, rigorous data collection methods, and comprehensive coverage of Fujian province, it also faces several limitations. It is important to note that a potential bias arises from the non-random selection of cities based on their economic development levels, which may not fully capture the diversity of the entire province. Although cities were randomly selected within each economic level, differences within cities of the same level might still affect the sample’s representativeness. Even though simple random sampling was used to select a secondary school and a high school from each administrative area, this approach might overlook unique local variations within the pre-selected areas and schools. Additionally, the third stage of cluster sampling, designed to ensure adequate representation of different grades, could still give rise to the underrepresentation or overrepresentation of certain classes, depending on the distribution of students within schools. Moreover, choosing a fixed percentage of classes within schools could lead to unequal representation in smaller or larger schools. These biases may limit the generalizability of the study’s findings to all adolescents in Fujian Province. It is also important to note that the reliance on self-reported data introduces the possibility of recall bias. Furthermore, as a cross-sectional study, it cannot establish causality between variables.

Although a multistage stratified cluster sampling design was used, the regression analyses did not explicitly account for clustering or sampling weights. As a result, the standard errors may have been slightly underestimated. Consequently, the reported standard errors and statistical significance may have been affected, and the findings should therefore be interpreted with appropriate caution. Future studies should employ survey-weighted analyses or multilevel modelling approaches that explicitly account for clustering and the complex sampling design. Another important limitation is that screen time was assessed as total daily screen time without distinguishing the duration spent on academic and recreational activities. Although participants indicated the reasons for using electronic devices, the time allocated to each activity was not quantified. Consequently, adolescents with similar total screen time may have differed substantially in their patterns of screen use, introducing exposure misclassification. This may have diluted or obscured activity-specific associations, particularly if recreational screen use has stronger relationships with sleep quality and psychological distress than educational screen use. Therefore, the findings should be interpreted as reflecting overall screen exposure rather than the health effects of specific screen-based activities. Future studies should quantify the duration of different screen activities separately to better disentangle these associations.

In addition, the possibility of residual confounding cannot be excluded. Although the analyses adjusted for several demographic and family-related characteristics, including age, sex, study grade, parental education, and time spent with parents, other factors that may influence both screen time and psychological health or sleep quality were not assessed. These include physical activity, obesity, caffeine or energy drink consumption, chronotype, family functioning, parent-child relationship quality, previous psychiatric disorders, medication use, alcohol or tobacco use, and other lifestyle-related factors. Consequently, the observed associations may be influenced by unmeasured confounding and should be interpreted with caution. Screen time should therefore be considered as one component within a broader biopsychosocial framework rather than the sole determinant of adolescent psychological well-being or sleep quality. Future studies, particularly longitudinal studies, should incorporate these additional variables to better account for potential confounding and to clarify the independent contribution of screen time.

Although 58,381 questionnaires were initially collected, only 48,432 complete responses were included in the final analyses following data screening. Because information on the characteristics of excluded participants was unavailable, a formal assessment of potential non-response or exclusion bias could not be performed. Consequently, the possibility of selection bias cannot be entirely excluded. The model yielded an adjusted R^2^ of 0.069, indicating that the combined demographic and psychological predictors account for 6.9% of the variance in adolescents’ screen time behavior. Critically, while the large sample size yields highly significant *p*-values, all individual predictor effect sizes fell below the f^2^ < 0.02 threshold. This indicates that while these factors are statistically detectable predictors, their isolated practical impact on screen time is negligible, suggesting that broader unexamined systemic, peer, or environmental factors dominate adolescent screen time behavior.

## Conclusion

The findings of this cross-sectional study indicate that screen time was significantly associated with poor sleep quality and psychological distress among adolescents. Although poor sleep quality showed the largest association with screen time among the variables examined, the overall explanatory power of the model was modest, suggesting that additional factors may also contribute to adolescents’ screen time behavior. Significant associations were also observed between screen time and demographic characteristics, including gender, age, and parental education. Male adolescents and older age groups were more likely to report higher screen time, whereas higher parental education levels were associated with lower screen time. As the screen time measure in this study represented overall daily screen exposure, including both educational and recreational activities, these findings should not be interpreted as indicating that all screen use is harmful. Rather, they highlight the importance of promoting healthy and balanced screen-use behaviors while recognizing the essential role of digital technologies in education. These findings highlight the importance of considering adolescents’ screen use alongside sleep quality, psychological health, and family characteristics in health promotion. Screen time should therefore be considered as one component within a broader biopsychosocial framework influencing adolescent health, rather than the sole determinant of sleep quality and psychological well-being. Future research should distinguish between academic and recreational screen use, as well as patterns of screen use such as nighttime exposure, to better understand which aspects of screen behavior are most strongly associated with adolescent health outcomes. Given the cross-sectional design of this study, the observed associations should not be interpreted as evidence of temporal or causal relationships. Longitudinal studies are warranted to confirm these associations and better understand the directionality of these relationships.

## Data Availability

The raw data supporting the conclusions of this article will be made available by the authors, without undue reservation.

## Supplementary material

Supplementary Figure 1 Proportion PSQI scores ≥ 5 across cities in Fujian Province.

Supplementary Figure 2 Proportion > 4 h of daily screen time across cities in Fujian Province.

Supplementary Appendix 1 Survey questionnaire.
