# Supplementary material for: Screen time and its association with psychological wellbeing and sleep quality: overview of the Fujian Adolescent Mental Wellness Study (FAMWeS)
Source: Front Psychol. 2026 Sep 11;17:1686399. doi: 10.3389/fpsyg.2026.1686399 (PMC13612499; doi:10.3389/fpsyg.2026.1686399)
Supplement: Supplementary file 1 [file Data_Sheet_1.pdf]

Your School：__________________； Student Number: _____________；

Age：_________； Grade：_____________； Classes：________；

Module 1: Basic Information

1.1 Your Gender： ☐ **Male** ☐ **Female**

1.2 You are from： ☐ **Rural** ☐ **Town** ☐ **City**

1.3 What grade level is your current academic performance in your year group?

☐ **Top 15% (class A)** ☐**15%-30% (class B)** ☐**30%-50% (class C)** ☐**50%-75% (class D)**

☐ **After 75% (class E)**

1.4 Do you feel pressured to learn? (**If "No", please skip to "1.5"**) ☐ **Yes** ☐ **No**

1.5 If "Yes", where does your stress come from?

☐**Parents** ☐**Teacher** ☐**Students** ☐**Others**

1.6 The composition of your family is？

☐ **Intact Families** ☐ **Single-parent families** ☐ **Divorced families** ☐ **Widowed families**

☐ **Intergenerational families**

1.7 What do you think your parents' parenting style is?

☐ **Didactic** ☐ **Consultative-democratic** ☐ **Autocratic coercive** ☐ **Spoiled** ☐ **Unclear**

1.8 Does your father spend much time with you?

☐ **Less than 1 hour/day** ☐**1-2 hours/day** ☐**2-3 hours/day** ☐**More than 3 hours/day**

☐ **Unaccompanied**

1.9 What does your father do with you every day?

☐ **Watch Movie** ☐ **Play Games** ☐ **Dinner and Chat** ☐ **Do Homework**

☐ **Parent-Child Reading** ☐ **Physical Exercise** ☐ **Otherwise**

1.10 Does your mother spend much time with you?？

☐ **Less than 1 hour/day** ☐**1-2 hours/day** ☐**2-3 hours/day** ☐**More than 3 hours/day**

☐ **Unaccompanied**

1.11 What does your mother do with you every day?

☐ **Watch Movie** ☐ **Play Games** ☐ **Dinner and Chat** ☐ **Do Homework**

☐ **Parent-Child Reading** ☐ **Physical Exercise** ☐ **Otherwise**

1.12 Your mother's level of education?

☐ **Unclear** ☐ **Elementary School** ☐ **Junior High School** ☐ **High School or Junior College**

☐ **Post-Secondary** ☐ **Undergraduate** ☐ **Postgraduates**

1.13 Your father's level of education?

☐ **Unclear** ☐ **Elementary School** ☐ **Junior High School** ☐ **High School or Junior College**

☐ **Post-Secondary** ☐ **Undergraduate** ☐ **Postgraduates**

1.14 Who do you currently live with?

☐ **Parents** ☐ **Father** ☐ **Mother** ☐ **Grandparents** ☐ **Other**

1.15 Do you have siblings? (**If "No", please skip to "1.16"**) ☐**Yes** ☐**No**

1.16 If "yes", you do?

☐ **older brother** ☐ **older sister** ☐ **younger brother** ☐ **younger sister**

1.17 Do you enjoy your time at school?

☐ **Very Enjoyable** ☐ **Enjoyment** ☐ **Sometimes Enjoy** ☐ **Not Enjoy**

☐ **Very Unenjoyable**

1.18 Do you like your classroom teacher?

☐ **Very Like** ☐ **Like** ☐ **Some Like** ☐ **Dislike** ☐ **Very Disliked**

1.19 Have you been bullied or teased by your classmates at school? ☐**Yes** ☐**No**

1.20 Do you bully or tease other students at school? ☐**Yes** ☐**No**

1.21 Do you feel safe at school? ☐ **Safety** ☐ **General** ☐ **Unsafe**

1.22 Do you have a religious affiliation? (**If "No", please skip to "1.23-1.26"**) ☐ **Yes** ☐**No**

1.23 What is your religion?

☐ **Buddhism** ☐ **Daoism** ☐ **Catholicism** ☐ **Christianity** ☐ **Islam** ☐ **Other**

1.24 When did you become religious?

☐ **pre-primary** ☐ **Primary stage** ☐ **Secondary school stage**

1.25 Do you participate in religious activities? (**If "No", please skip to "1.26"**) ☐**Yes** ☐**No**

1.26 How many times a week do you attend religious services?

☐**1** ☐**2** ☐**3** ☐**4** ☐**5 or more**

1.27 Are your parents or other family members also religious?

☐ **Father's Faith** ☐ **Mother's Faith** ☐ **Both parents believe** ☐ **Other members' beliefs**

☐ **Nobody believes**

1.28 Do you participate in physical activity or sports on a weekly basis outside of school hours?

☐**No** ☐**1 time/week** ☐**2-3 times/week** ☐**4-5 times/week** ☐**More than 5 times/week**

1.29 How long do you engage in physical exercise or physical activity each time?

☐**No** ☐**30 minutes or less** ☐**Within 1 hour** ☐**1-2 hours** ☐**More than 2 hours**

1.30 What sports or physical activities do you typically participate in?

☐ **Big ball (soccer, basketball, volleyball, etc.)** ☐ **Small ball (badminton, table tennis, tennis, etc.)** ☐ **Physical fitness (aerobics, dance, yoga, etc.)** ☐ **Athletics (running, long jump, etc.)** ☐ **Basic physical fitness classes (jumping rope, swimming, etc.)** ☐ **Chess (Xiangqi, Weiqi, etc.)** ☐ **Snow and ice (skating, skiing, etc.)** ☐**Combat classes (martial arts, boxing, fighting, etc.)** ☐ **Others**

1.31 Do you have or have you ever idolized? (**If "No", please skip to "1.32-1.33"**) ☐ **Yes** ☐**No**

1.32 How long have you been idolizing?

☐ **Beginning** ☐**6 months** ☐**12 months** ☐**1 to 3 years** ☐ **More than 3 years**

1.33 Who are your idols? (**multiple**)

☐ **Political and military leaders** ☐ **Business Elite Entrepreneurs** ☐ **Heroic and typical figures in society** ☐ **Writers, artists** ☐ **Eminent scholars and thinkers** ☐ **Movie and TV stars** ☐ **sports star** ☐ **A close parent, relative, teacher or friend** ☐ **Grassroots star** ☐ **religious figure** ☐ **Avatar** ☐ **oneself** ☐ **Others**

1.34 Are you enrolled in an off-campus tutoring program? (**If "No", please skip to "1.35"**)

☐**Yes** ☐**No**

1.35 The off-campus tutoring programs you participate in are?

☐ **Culture (math, English, etc.)** ☐ **Arts (musical instruments, calligraphy, etc.)**

☐ **Science (computers, robotics, etc.)** ☐ **Sports (badminton, basketball, etc.)**

☐ **Competitions (Olympiads, etc.)** ☐ **Others**

1.36 Do you usually have discretionary and activity time? (**If "No", please skip to "1.37"**) ☐**Yes** ☐**No**

1.37 What do you do in your free time?

☐ **Learn** ☐ **Play games** ☐ **Go out and play with your classmates** ☐ **Sports**

☐ **Solitude** ☐ **Others**

1.38 Do you use computers, cell phones, and other electronics? (**If "No", please skip to "1.39-1.40"**)

☐**Yes** ☐**No**

1.39 What do you use electronics for?

☐ **Learn** ☐ **Look up information** ☐ **Video entertainment** ☐ **Play games** ☐ **Others**

1.40 How many hours a day do you use electronics?

☐**<30 min** ☐**30 min-1 hour** ☐**1 hr-4 hrs** ☐**>4 hours.**

1.41 Does your school have a psychologist? ☐ **Yes** ☐**No** ☐ **Don't know**

1.42 Does your school have a mental health school-based program?

☐ **Yes** ☐**No** ☐ **Don't know**

1.43 How many times a week is your school's mental health school-based program？

☐**1** ☐**2** ☐**3** ☐**4** ☐ **More than 5** ☐**No**

1.44 Do you have your own hobbies? ☐**No** ☐**Yes**，Please write your hobbies___________________

Module 2: Overall Mental Health Status

In this module we will find out about your most recent health conditions, these questions address conditions **from 2 or 3 weeks ago to the present**, please tick the column you most closely match.

Items	never	few	sometimes	often
2.1 Can you focus on whatever you're doing?				
2.2 Insomnia due to anxiety				
2.3 Feeling like playing a role in things?				
2.4 Feeling able to make decisions about things?				
2.5 I feel nervous all the time.				
2.6 I feel like I can't get over it.				
2.7 Can you enjoy the daily activities?				
2.8 Can you face the conflict?				
2.9 Feeling upset and depressed				
2.10 Lost confidence in yourself?				
2.11 Consider yourself a worthless person				
2.12 Overall, feeling moderately happy?				

Module 3: Emotional States

We would like to find out how you have been feeling in THE PAST WEEK. There are some sentences below. Please circle the number which best shows how TRUE each sentence was of you **during the past week.** There are no right or wrong answers.

Items	Not True	A Little True	Fairly True	Very True
3.1 I got upset about little things				
3.2 I felt dizzy, like I was about to faint				
3.3 I did not enjoy anything				
3.4 I had trouble breathing (e.g. fast breathing), even though I wasn't exercising and I was not sick				
3.5 I hated my life				
3.6 I found myself over-reacting to situations				
3.7 My hands felt shaky				
3.8 I was stressing about lots of things				
3.9 I felt terrified				
3.10 There was nothing nice I could look forward to				
3.11 I was easily irritated				
3.12 I found it difficult to relax				
3.13 I could not stop feeling sad				
3.14 I got annoyed when people interrupted me				
3.15 I felt like I was about to panic				
3.16 I hated myself				
3.17 I felt like I was no good				
3.18 I was easily annoyed				
3.19 I could feel my heart beating really fast, even though I hadn't done any hard exercise				
3.20 I felt scared for no good reason				
3.21 I felt that life was terrible				

Module 4: Sleep conditions

In this module, we will find out about your recent sleep status and sleep quality. The following questions are about your sleep **in the last 1 month**, please select or fill in the answer that best fits your situation.

4.1 During the past month, what time have you usually gone to bed at night?

☐ After 11 p.m. ☐ 10-11 p.m. ☐9-10 p.m. ☐Before 9 p.m.

4.2 During the past month, how long (in minutes) has it usually taken you to fall asleep each night?

☐ ≤15 min. ☐15 - 30 min. ☐30-60 min. ☐≥60 min.

4.3 During the past month, what time have you usually gotten up in the morning?

☐ Before 6:00 a.m. ☐6-7 a.m. ☐7-8 a.m. ☐9-10 a.m. ☐ After 10:00 a.m.

4.4 During the past month, how many hours of **actual sleep** did you get at night? (This may be different than the number of hours you spent in bed.)

☐ ≥ 7 hours ☐ 6-7 hours ☐ 5-6 hours ☐ <5 hours or more

For each of the remaining questions, check the one best response. Please answer all question

4.5 During the past month, how often have you had trouble sleeping because you……

	Not	<4 times	4-8times	≥ 9times
4.5.1 Cannot get to sleep within 30 minutes				
4.5.2 Wake up in the middle of the night or early morning				
4.5.3 Have to get up to use the bathroom				
4.5.4 Cannot breathe comfortably				
4.5.5 Cough or snore loudly				
4.5.6 Feel too cold				
4.5.7 Feel too hot				
4.5.8 Had bad dreams				
4.5.9 Have pain				
4.5.10 Other reason(s)				

Please describe：

4.6 During the past month, how would you rate you sleep quality overall?

☐Very good ☐Fairly good ☐Fairly bad ☐Very bad

4.7 During the past month, how often have you taken medicine to help you sleep (prescribe or “over the counter)

☐Not ☐Less than once a week ☐Once or twice a week ☐Three or more times a week

4.8 During the past month, how often have you had trouble staying awake while driving, eating meals, or engaging in social activity

☐Not ☐Less than once a week ☐Once or twice a week ☐Three or more times a week

4.9 During the past month, how much of a problem has it been for you to keep up enthusiasm to get things done?

☐Not ☐Sometimes ☐Often ☐Always
